# Factors related to pregnancy status and unwanted pregnancy among lebanese women during the COVID-19 lockdown: a cross-sectional study

**DOI:** 10.1186/s13690-022-00833-2

**Published:** 2022-02-25

**Authors:** Chadia Haddad, Sandrella Bou Malhab, Hala Sacre, Diana Malaeb, Joelle Azzi, Dalia Khachman, Nathalie Lahoud, Pascale Salameh

**Affiliations:** 1 Research department, Psychiatric Hospital of the Cross, Jal Eddib, Lebanon; 2 INSPECT-LB (Institut National de Santé Publique, d’Épidémiologie Clinique Et de Toxicologie-Liban), Beirut, Lebanon; 3 Faculté de santé, Université Sainte Famille, Batroun, Lebanon; 4 School of Pharmacy, Lebanese International University, Beirut, Lebanon; 5 College of Pharmacy, Gulf Medical University, Ajman, United Arab Emirates; 6 Faculty of Public Health, Lebanese University, Fanar, Lebanon; 7 Clinical and Epidemiological Research Laboratory, Faculty of Pharmacy, Lebanese University, Hadat, Lebanon; 8 Faculty of Pharmacy, Lebanese University, Beirut, Lebanon; 9 University of Nicosia Medical School, Nicosia, Cyprus

**Keywords:** Women, Pregnancy, COVID-19, Unwanted pregnancy, Lockdown

## Abstract

**Background:**

Home confinement and lockdowns have created challenges and vulnerabilities, causing relevant changes in sexual health and couple stability, particularly in women. The objective of this study was to evaluate the socio-economic and psychological factors related to current pregnancy status and unwanted pregnancy among Lebanese women during the COVID-19 lockdown.

**Methods:**

A cross-sectional online study conducted between June 8 and August 1, 2020, enrolled 369 Lebanese women using the snowball technique for data collection. All married women between 18 and 51, with access to the internet and currently living with their partners, were eligible to participate. Current pregnancy status and unwanted pregnancy were assessed using binary questions. The SPSS software 25 was used for data analysis, and multivariable analysis was performed, taking the pregnancy status and unwanted pregnancy as the dependent variables. The statistical significance was set at a *p*-value < 0.05.

**Results:**

Our results showed that 11.1% of women were pregnant, of whom 22.0% reported unwanted pregnancies. Having children (ORa = 0.183) and taking contraceptives (ORa = 0.231) were significantly associated with a reduced chance of becoming pregnant. Higher psychological violence would negatively affect pregnancy, but the association was not significant (*p* = 0.065). Regular visits to the physician for routine checks were also linked to a decreased risk of unwanted pregnancy (ORa = 0.053). Higher psychological violence would affect unwanted pregnancy; however, the association was insignificant (*p* = 0.056).

**Conclusion:**

The study findings showed that having children and taking contraceptives are associated with a reduced pregnancy. Additionally, psychological violence was found to be related to current pregnancy status and unplanned pregnancy. During a pandemic, vulnerable women should be identified and given adequate care, knowledge, and awareness regarding their reproductive health.

**Supplementary Information:**

The online version contains supplementary material available at 10.1186/s13690-022-00833-2.

## Background

The coronavirus disease 2019 (COVID-19), a viral respiratory illness caused by the novel severe acute respiratory syndrome coronavirus-2 (SARS-CoV-2), has so far affected 220 countries, resulting in 177 million infections and 3.0 million deaths (as of June 2021) [1]. The best strategy to prevent the spread of the disease is through public health measures, including confinement and social distancing.

While most of the world is settling into a new social distancing pattern, couples are likely to spend more free time together at home, leading to more intimacy [2]. Some couples who had planned to have a child before the COVID-19 pandemic are still attempting; others even started to express their desire for parenthood during confinement [2]. However, confinement and lockdowns have created challenges and vulnerabilities, particularly in women, causing relevant changes in sexual health and couple stability [3]. The vulnerabilities are related to social, political, and economic systems, which in turn magnify the pandemic effects [4]. Specifically, during lockdowns, there are wide discrepancies in the sexual desire within couples, where some use sex as a coping mechanism to stay connected and relieve anxiety while others completely lose interest in sex [5]. In Italy, a study has shown that stress at work and the bustle of everyday life can be potent sexual inhibitors that lead to a lower propensity to get pregnant due to concerns about future economic hardships and the potential consequences of the disease on pregnancy [2].

On the other hand, lockdown and minimal contact with the outside community trigger all types of violence [6]. Indeed, incidents of violence against women have dramatically increased worldwide since countries implemented lockdowns to contain the pandemic [7]. While data on the current COVID-19 lockdown situation is limited, studies of past natural disasters and their effects highlight predictors of increased violence during these periods. Violence at home is strongly associated with the male partner. Loss of income for male partners creates a lower degree of control over economic security and exerts more control over their partners. Individuals may resort to transactional sex to meet their basic needs and cope with reduced and inadequate income, which may increase the risk of unwanted pregnancies [8], with several studies highlighting the strong association between partner violence and unwanted pregnancy [9, 10]. Abused women must compromise about contraceptive or condom use and family planning [11, 12]. Their lack of control over their reproductive health is increasingly recognized as a critical mechanism underlying a high risk of unwanted pregnancy, further increased during certain pandemic conditions, such as COVID-19. Many reasons trigger the development of unwanted pregnancy, including forced sex, financial difficulties, having children, and being unmarried [13]. Preliminary reports on COVID-19 indicate increased unplanned and unwanted pregnancies due to rapidly dwindling stocks of contraceptives, increased incidence of domestic violence, and growing income insecurity [14].

There is currently little information about pregnancy status and unwanted pregnancy during the COVID-19 epidemic. Therefore, this study aimed to evaluate socioeconomic and psychological factors related to current pregnancy and unwanted pregnancy among a sample of Lebanese women during the COVID-19 lockdown.

## Methods

### Study design and sampling

A cross-sectional online study conducted between June 8 and August 1, 2020, enrolled a sample of 369 Lebanese women using the snowball technique for data collection. All married women between 18 and 51 with internet access and currently living with their partners were eligible to participate. Excluded were those with a fertility problem and those single, widowed, or divorced. The questionnaire developed on Google Forms was distributed through social media and WhatsApp groups and required 40 min to complete. Inclusion criteria were available in the consent form at the beginning of the survey. Participation in this study was voluntary, and participants received no compensation in return. The anonymity of participants was guaranteed during the data collection process.

### Minimum sample size calculation

The Epi info software (Centers for Disease Control and Prevention, Epi Info™) calculated a minimum sample of 306 participants, considering a Lebanese female population of 2,294,260 [15], a prevalence of 15% of pregnant women [16], 95% confidence level, and after adding a margin of error of 4%. A sample of 500 women was targeted to allow for missing values. The final sample size included 369 participants.

### Translation and piloting

The online survey consisted of closed-ended questions in English and Arabic. It was pilot tested on ten subjects to check the clarity of the questionnaire; related data were included in the final dataset. The link to Google Forms was then distributed to potential respondents.

A forward and backward translation was conducted for all the items of the questionnaire. One translator was in charge of translating the scales from English into Arabic, and a second one performed the back translation. Discrepancies between the original English version and the translated one were resolved by consensus.

### Questionnaire

The questionnaire consisted of three sections. The first one assessed the sociodemographic details of participants (age, educational level, the region of residence, religion, working status, monthly income, smoking and alcohol status, and physical activity) in addition to the sociodemographic characteristics of the partner as reported by the participant woman herself. The household crowding index was calculated by dividing the number of persons living in the house by the number of rooms, excluding the bathroom and kitchen [17]. The monthly income was divided into four levels: no income, low < 1,000 USD, intermediate 1,000–2,000 USD, and high income > 2,000 USD. Moreover, fear of poverty was measured on a Likert scale from 0 to 10, where zero indicates no fear of poverty and ten extreme fear of poverty.

The second section consisted of questions selected from other studies [18–20] and constructed by the authors based on the research questions. The questions focused on the couple and the children, the woman's role in the family, the woman's relationship with her partner, in addition to items related to pregnancy status and concerns during the lockdown. Examples of the asked questions: “Do you discuss family planning with your partner?”, “Are you capable of meeting the financial needs of your family?”, “Have you ever faced any pregnancy-related complications (such as severe bleeding, unsafe abortion)?”, “Do you have a history of negative pregnancy outcomes (such as a neonatal death, miscarriage, or stillbirth)”, “How do you describe your current pregnancy?” and “Were you regularly visiting your doctor for routine checkups during the lockdown?”. The current pregnancy status and unwanted pregnancy were assessed using binary questions (Yes/No). Pregnancy status was defined as being “pregnant” or “not”.

The last part consisted of a scale to measure violence, the Composite Abuse Scale (Revised) – Short Form (CASR-SF). This 15-item scale covers three domains, i.e., physical, sexual, or psychological abuse; it evaluates exposure and abuse frequency [21]. The total score is calculated by summing the 15 responses. Items are graded on a Likert scale from 1 to 6, where a higher score indicates a higher intensity/occurrence of abuse. Three subscales scores are derived from the total score, reflecting physical (4 items), sexual (2 items), and psychological (6 items) abuse [21]. In this study, Cronbach’s alpha was 0.902 for the total scale, 0.791 for the psychological abuse subscale, 0.759 for the physical abuse subscale, and 0.740 for the sexual abuse subscale. The author of the questionnaire, Professor Marilyn Ford-Gilboe, granted permission to use the scale.

### Statistical analysis

Completed forms were imported into a Microsoft Excel spreadsheet. Data were then analyzed on Statistical Package for the Social Sciences (SPSS) software version 23 (Chicago, IL, USA). A descriptive analysis was performed using the counts and percentages for categorical variables and means and standard deviations for continuous measures. The Student’s t-test and Chi-Square test were used to compare means and frequencies between the different subgroups, respectively, to assess the association between variables; assumptions of continuous variables normality and other conditions were checked. When conditions were not fulfilled, the Mann Whitney and the Fisher exact test were used, respectively. Two logistic regressions using the forward method were performed: the first took the pregnancy status as the dependent variable, and the other the wanted/unwanted pregnancy as the dependent variable. The variables in the bivariate analysis that showed a *p*-value less than 0.05 were used in the multivariable analysisto minimize confounding. The statistical significance was set at a *p*-value < 0.05.

## Results

### Sample description

Table 1 presents details regarding the sociodemographic and other characteristics of the sample. The mean age of women was 32.5 ± 6.4 years, the majority had a university education level (87.5%), 59.9% were employed, 27.6% had no income, and 42.5% practiced physical activities. Only 31.2% of them were smokers, and 10.8% consumed alcohol.

**Table 1:** Sociodemographic and other characteristics of the studied sample (*N* = 369)

	**Woman response**	**Partner characteristics reported by woman**
	**Frequency (%)**	**Frequency (%)**
**Education level**		
Primary	3 (0.8%)	18 (4.9%)
Complementary	11 (3.0%)	34 (9.2%)
Secondary	32 (8.7%)	64 (17.3%)
University	323 (87.5%)	253 (68.6%)
**Religion**		
Christian	115 (31.2%)	115 (31.2%)
Muslim	155 (42.0%)	159 (43.1%)
Druze	81 (22.0%)	80 (21.7%)
Atheist	2 (0.5%)	2 (0.5%)
Refused to answer	16 (4.4%)	13 (3.5%)
**Working status**		
Employed	221 (59.9%)	334 (90.5%)
Unemployed	148 (40.1%)	35 (9.5%)
**Monthly income**		
No income	102 (27.6%)	19 (5.1%)
Low	94 (25.5%)	71 (19.2%)
Intermediate	112 (30.4%)	168 (45.5%)
High	61 (16.5%)	111 (30.1%)
**Smoking status**		
Non smoker	254 (68.8%)	170 (46.1%)
Smoker	115 (31.2%)	199 (53.9%)
**Alcohol consumption**		
Yes	40 (10.8%)	148 (40.1%)
No	329 (89.2%)	221 (59.9%)
**Physical activity**		
Yes	157 (42.5%)	128 (34.7%)
No	212 (57.5%)	241 (65.3%)
	**Mean ± SD**	**Mean ± SD**
**Age in years**	32.5 ± 6.4	37.6 ± 7.2
**Days of lockdown**	71.0 ± 42.8	
**Fear of poverty**	5.8 ± 3.2	

Also, the majority of partners had a university education level (68.6%) and were employed (90.5%); 45.5% had an intermediate income level, 53.9% were smokers, 40.1% consumed alcohol, and 34.7% practiced physical activities. The mean age of the partners was 37.6 ± 7.2 years.

The mean duration of lockdown was 71.0 ± 42.8 days, and the mean fear of poverty was 5.8 ± 3.2.

### Reproductive status of women

The mean duration of marriage was 7.8 ± 5.9 years, and the mean number of pregnancies was 2.1 ± 1.5. Only 24.7% of women had a history of negative pregnancy outcomes, 7.9% had a history of pregnancy termination, 16.8% had a history of unintended pregnancy, and 16.0% had pregnancy-related complications. Table 2 shows the other characteristics of women’s reproductive status (Table 2).

**Table 2:** Reproductive status of the study participants

	**Frequency (%) *N* = 369**
**History of pregnancy negative outcome**	91 (24.7%)
**History of pregnancy termination**	29 (7.9%)
**History of unintended pregnancy**	62 (16.8%)
**Pregnancy related complications**	59 (16.0%)
**Frequency of sexual intercourse**	
Four to eight in four weeks	167 (45.3%)
Equal to or more than 12 in four weeks	80 (21.7%)
One to three in four weeks	88 (23.8%)
None in four weeks	34 (9.2%)
**Discussion with partner about family planning**	
Never	9 (2.5%)
Sometimes	99 (26.8%)
Always	261 (70.7%)
**Couple wants the same number of children**	316 (85.6%)
**Fertility preferences for the woman**	
Desire to have children	192 (52.0%)
Desire to stop childbearing	177 (48.0%)
	**Mean ± SD**
**Duration of marriage in years**	7.8 ± 5.9
**Age at marriage in years**	24.8 ± 3.9
**Number of pregnancies**	2.1 ± 1.5
**Number of children**	2.0 ± 0.85
**Age at first birth**	26.0 ± 5.2

### Bivariate analysis: correlates of pregnancy status

Of the total sample, 41 women (11.1%) were pregnant. As compared to pregnant women, non-pregnant women had a significantly higher mean age (*M*_non-pregnant_ = 32.9 vs. *M*_pregnant_ = 29.1, *p* < 0.001), number of children (*M*_non-pregnant_ = 2.0 vs. *M*_pregnant_ = 1.5, *p* < 0.001), psychological violence (*M*_non-pregnant_ = 1.4 vs. *M*_pregnant_ = 0.29, *p* < 0.001), physical violence (*M*_non-pregnant_ = 0.42 vs. *M*_pregnant_ = 0.10, p = 0.005), sexual violence (*M*_non-pregnant_ = 0.17 vs. *M*_pregnant_ = 0.00, *p* < 0.001), and total violence (*M*_non-pregnant_ = 2.2 vs. *M*_pregnant_ = 0.39, *p* < 0.001). Additionally, a significantly higher mean partner age (*M*_non-pregnant_ = 38.05 vs. *M*_pregnant_ = 33.83, *p* < 0.001) was found in non-pregnant women as compared to pregnant women. A significantly higher proportion of pregnant women had never worked (17.1%), were former smokers (42.9%), had no children (25.8%), wanted to have children (16.1%), had no history of child (12.5%) and partner abuse (13.5%), and did not use contraception (31.7%). Regarding partners, a significantly higher proportion of those with high income (18.0%), as compared to the other categories, had a pregnant woman partner. No significant association was found for the other used variables (Supplementary Table 1).

### Bivariate analysis: correlates of unwanted pregnancy

A significantly higher proportion of unwanted pregnancies was found among women with a history of unwanted pregnancy (80.0%), who worked from home (54.5%), sometimes discussed with their partner about family planning (50.0%), desired to stop childbearing (50.0%), and did not regularly visit their physician (57.1%). The association between the violence scale and subscales and unwanted/wanted pregnancy was not significant. Similarly, no significant association was found for the other used variables (Supplementary Table 2).

### Multivariable analysis

A first logistic regression taking current pregnancy during the lockdown as the dependent variable showed that women who had children (ORa = 0.183) and used contraceptives (ORa = 0.231) were at lower odds of getting pregnant. The association between psychological violence and pregnancy tended toward significance (*p* = 0.065) (Table 3, Model 1).

**Table 3:** Multivariable analysis

**Model 1: Logistic regression taking the pregnancy status during quarantine as dependent variable**						
**Factor**		**ORa**	**95% CI**			***p*-value**
Having children		0.183	0.074; 0.452			< 0.001
Contraception use before getting pregnant vs. no use^a^		0.231	0.063; 0.853			0.028
Psychological violence		0.738	0.534; 1.020			0.065
Variables entered: having children, fertility preferences, partner’s age, partner’s income, woman’s age, woman’s work status during quarantine, woman’s smoking status and contraception use, psychological, physical and sexual violence						
**Model 2: Logistic regression taking the wanted/unwanted pregnancy as the dependent variable**						
**Factor**	**ORa**			**95% CI**	***p*-value**	
Regularly visiting physician for routine checkup	0.053			0.005; 0.515	0.011	
Psychological violence	4.482			0.963; 20.861	0.056	

Variables entered: past unintended pregnancy, family plan, fertility preferences, visiting regularly the physician, psychological, physical and sexual violence

^a^Reference group; *CI*, confidence interval, *ORa* adjusted odds ratio

A second logistic regression taking the wanted/unwanted pregnancy as the dependent variable showed that regular visits to the physician for routine checkups (ORa = 0.053) were significantly associated with lower odds of unwanted pregnancy. The association between psychological violence and unwanted pregnancy tended toward significance (*p* = 0.056) (Table 3, Model 2).

## Discussion

To the best of our knowledge, this study is the first to assess the factors correlated with current pregnancy status and unwanted pregnancy during the COVID-19 lockdown among 369 Lebanese women. Our results showed that 11.1% of women were pregnant, of whom 22.0% reported unwanted pregnancies. Recently, several large-scale studies explored pregnancy and fertility during the coronavirus pandemic, but none has published yet. Also, available data on the impact of a disaster on reproduction and fertility are contradictory. Some studies reported increased birth rates following short-term disasters [22, 23], while others showed a decrease in pregnancies [24, 25]. Globally, coronavirus affects the reproductive choice of both those who choose to conceive and those who do not. Indeed, worrying thoughts surrounding this pandemic may influence sexual activity, and employment loss and economic instability may lead to a delay in pregnancy. Thus, there will still be individuals who cannot regulate their reproductive decisions. Also, women planning to conceive will be concerned about the care and treatment they get during pregnancy while in lockdown. The prevalence of unwanted pregnancies in this study is lower than that reported in other countries (ranging from 26 to 50%) [26–33], which may not reflect the actual rate of unwanted pregnancies in the Lebanese community, as our sample may not be representative of the entire population of pregnant women in Lebanon.

Our study showed that having already children and using contraceptives are associated with a lower probability of pregnancy. Indeed, families with multiple children may consider any potential pregnancy as unwanted and rely on birth control methods to prevent pregnancy [34–37]. Educated and empowered women are also more likely to take charge of their reproductive health and household management [38–40]; they would refuse to have forced intercourse or unwanted pregnancy, using contraception methods to avoid further childbearing and unsafe abortion [41–43]. Additionally, women of advanced maternal age, with adequate education and free from financial constraints, have more access to contraceptives than their peers [44, 45]. Several factors encourage couples to use family planning effectively, including regular sexual activity, good communication between partners regarding their reproductive health and child-rearing, financial hardship, and job instability [46]. Thus, older couples and well-educated spouses engage in fertility regulation interventions, which reduces unwanted pregnancies and induced abortions and contributes to small family size [40, 47, 48].

Findings from this study have shown that women who have been subjected to psychological violence may have a lesser chance of becoming pregnant and have an undesirable pregnancy, in line with previous results showing a strong correlation between domestic violence and unintended pregnancy [49–51]. In a recent study conducted in Ireland during the COVID-19 pandemic among 70 women, 4.3% reported relationship deterioration with their partners, and 11% stated that there were tensions between family members confined in the same household [52]. The COVID-19 lockdown has raised many challenges such as stress, anxiety, financial difficulties, and social isolation that have impacted the mental health status of women and their relationship with their partners and family members; such conditions can aggravate the risk of violence in families and worsen situations at homes [53]. Although not conclusively established, experiences of violence may also be associated with risky behaviors and poor pregnancy outcomes [54]. It is essential to document the long-term physical and psychological impacts of abuse and the role these interactions can play in rising stress associated with a spectrum of detrimental health outcomes [54]. Moreover, it is unclear whether the lockdown has a negative or positive impact on the mental health status of women and how the severity or frequency of violence experienced by abused women changes during pregnancy [52].

Our results revealed that women who visit their physician for routine checkups have a lower probability of unwanted pregnancy. Similarly, previous findings have shown that providing women with birth control techniques and counseling reduces unplanned pregnancies and abortions [55–57]. A prospective study of 10,000 reproductive-aged women found that structured counseling could improve knowledge and consistent use of contraceptive methods, which decreases the rate of unintended pregnancy, abortion, and births [58]. The unmet need for contraception, unreliable or inaccurate use of contraceptive methods, and misinformation about adverse effects, especially for hormonal or long-acting reversible contraceptives, are at the core of unwanted pregnancies [59]. Therefore, gynecologist counseling will provide women with sufficient information about the different contraceptive methods, including their effectiveness, availability, risks, and benefits, resulting in controlling birth and decreasing unwanted pregnancies [60]. During the COVID-19 pandemic, regular checkup visits may be delayed or interrupted to lower the risk of exposure to the virus, but telemedicine or any telehealth method could overcome this problem.

### Study implications

Our study highlights the need for active measures to monitor and manage violence as an indispensable part of the fight against COVID-19. Despite the provision of basic needs and promptly implemented actions to contain the pandemic, yet violence encountered during this period should be thoroughly addressed and investigated as it is associated with long-term devastating consequences.

### Limitation

This study has several limitations. Its results could not be generalized to the population because of the unequal sample enrollment and distribution across Lebanon. Also, its cross-sectional design cannot infer causality. Since it assessed adult women living with a partner, this study did not explore the adolescent pregnancy status. Information bias could also exist as the study questionnaire was online and answers were self-reported. Selection bias might have also occurred since the sample was not randomly selected but gathered using the snowball sampling technique. Most participants were well-educated, computer literate, and had internet access; thus, less-educated people and those with no internet access could not be assessed. Residual confounding bias is also possible since there might be factors related to pregnancy and unwanted pregnancy that were not measured in this study. Additionally, participants' privacy might have been breached as the questionnaire required 40 min to complete, which could have involved partner interference, especially in case of partner violence.

## Conclusion

Our main findings indicate that women who had children and used contraceptives had a lower likelihood of pregnancy. Furthermore, psychological violence tended to be an associated factor for current pregnancy status and unwanted pregnancy. Thus, vulnerable women should be identified and offered appropriate care, information, and awareness regarding their reproductive health during a pandemic.

## Supplementary Information

**Additional file 1: Supplementary Table S1. **Bivariate analysis taking the current pregnancy status (Yes (41 (11.1%)) / No (328 (88.9%)) as the dependent variable

**Additional file 2: Supplementary Table S2. **Bivariate analysis taking the wanted/unwanted pregnancy as the dependent variable (*N*=41)

## Data Availability

Data can be made available under reasonable request form the corresponding author.

## Abbreviations

SARS-CoV2: Severe acute respiratory syndrome coronavirus 2
COVID-19: Coronavirus disease 2019
USD: United States dollar
CASR-SF: The Composite Abuse Scale (Revised) – Short Form
SPSS: Statistical Package for Social Sciences
SD: Standard Deviation,
ORa: Adjusted odds ratios
CI: Confidence Interval
