# Supplementary material for: Factors related to pregnancy status and unwanted pregnancy among lebanese women during the COVID-19 lockdown: a cross-sectional study
Source: Arch Public Health. 2022 Feb 25;80:68. doi: 10.1186/s13690-022-00833-2 (PMC8874299; doi:10.1186/s13690-022-00833-2)
Supplement: Supplementary file 1 — Additional file 1: Supplementary Table S1. Bivariate analysis taking the current pregnancy status (Yes (41 (11.1%)) / No (328 (88.9%)) as the dependent variable [file 13690_2022_833_MOESM1_ESM.docx]

**Supplementary Tables:**

| **Supplementary Table 1: Bivariate analysis taking the current pregnancy status (Yes (41 (11.1%)) / No (328 (88.9%)) as the dependent variable** | | | | | | |
| --- | --- | --- | --- | --- | --- | --- |
|  | **Current pregnancy status** | | | | | |
|  | **Woman’s characteristics** | | | **Partner’s characteristics** | | |
|  | **No** | **Yes** | **p-value** | **No** | **Yes** | **p-value** |
|  | **Mean (SD)** | **Mean (SD)** |  | **Mean (SD)** | **Mean (SD)** |  |
| **Age in years** | 32.9 (6.4) | 29.1 (5.1) | **<0.001** | 38.1 (7.3) | 33.8 (5.4) | **<0.001** |
| **Age at marriage in years** | 24.8 (3.8) | 25.3 (4.1) | 0.381 | 30.6 (14.0) | 30.1 (4.6) | 0.793 |
| **Household crowding index** | 0.98 (0.44) | 0.87 (0.50) | 0.150 |  |  |  |
| **Fear of poverty** | 5.9 (3.1) | 5.3 (3.5) | 0.230 |  |  |  |
| **Number of children** | 2.0 (0.85) | 1.5 (0.66) | **<0.001** |  |  |  |
| **CASR psychological** | 1.4 (3.2) | 0.29 (0.84) | **<0.001** |  |  |  |
| **CASR physical** | 0.42 (1.7) | 0.10 (0.44) | **0.005** |  |  |  |
| **CASR sexual** | 0.17 (0.83) | 0.00 (0.00) | **<0.001** |  |  |  |
| **CASR total** | 2.2 (6.1) | 0.39 (1.2) | **<0.001** |  |  |  |
|  | **N (%)** | **N (%)** |  | **N (%)** | **N (%)** |  |
| **Work status** |  |  |  |  |  |  |
| *From home* | 117 (91.4%) | 11 (8.6%) | **0.019** | 68 (93.2%) | 5 (6.8%) | 0.282 |
| *Go to work* | 78 (83.9%) | 15 (16.1%) |  | 227 (87.0%) | 34 (13.0%) |  |
| *Unemployed* | 75 (96.2%) | 3 (3.8%) |  | 30 (93.8%) | 2 (6.3%) |  |
| *Never works* | 58 (82.9%) | 12 (17.1%) |  | 3 (100.0%) | 0 (0.0%) |  |
| **Monthly income** |  |  |  |  |  |  |
| *No income* | 89 (87.3%) | 13 (12.7%) | 0.194 | 17 (89.5%) | 2 (10.5%) | **0.048** |
| *Low income* | 87 (92.6%) | 7 (7.4%) |  | 66 (93.0%) | 5 (7.0%) |  |
| *Intermediate income* | 95 (84.8%) | 17 (15.2%) |  | 154 (91.7%) | 14 (8.3%) |  |
| *High income* | 57 (93.4%) | 4 (6.6%) |  | 91 (82.0%) | 20 (18.0%) |  |
| **Smoking status** |  |  |  |  |  |  |
| *Regular* | 39 (97.5%) | 1 (2.5%) | **<0.001** | 125 (91.9%) | 11 (8.1%) | 0.104 |
| *Occasional* | 69 (92.0%) | 6 (8.0%) |  | 51 (81.0%) | 12 (19.0%) |  |
| *Former smoker* | 8 (57.1%) | 6 (42.9%) |  | 19 (95.0%) | 1 (5.0%) |  |
| *Non-smoker* | 212 (88.3%) | 28 (11.7%) |  | 133 (88.7%) | 17 (11.3%) |  |
| **Type of smoking** |  |  |  |  |  |  |
| *Cigarette* | 21 (84.0%) | 4 (16.0%) | 0.089 | 73 (90.1%) | 8 (9.9%) | 0.753 |
| *Waterpipe* | 84 (96.6%) | 3 (3.4%) |  | 73 (90.1%) | 8 (9.9%) |  |
| *Cigarette and waterpipe* | 7 (100.0%) | 0 (0.0%) |  | 4 (100.0%) | 0 (0.0%) |  |
| *Other* | 0 (0.0%) | 0 (0.0%) |  | 8 (80.0%) | 2 (20.0%) |  |
| **Do you have children?** |  |  |  |  |  |  |
| *No* | 49 (74.2%) | 17 (25.8%) | **<0.001** |  |  |  |
| *Yes* | 279 (92.1%) | 24 (7.9%) |  |  |  |  |
| **Fertility preferences** |  |  |  |  |  |  |
| *Have more children* | 161 (83.9%) | 31 (16.1%) | **0.001** |  |  |  |
| *Stop childbearing* | 167 (94.4%) | 10 (5.6%) |  |  |  |  |
| **Past pregnancy complications** |  |  |  |  |  |  |
| *No* | 272 (87.7%) | 38 (12.3%) | 0.108 |  |  |  |
| *Yes* | 56 (94.9%) | 3 (5.1%) |  |  |  |  |
| **Frequency of sexual intercourse** |  |  |  |  |  |  |
| *1-2/wk* | 140 (83.8%) | 27 (16.2%) | 0.019 |  |  |  |
| *≥3/wk* | 74 (92.5%) | 6 (7.5%) |  |  |  |  |
| *1-2/month* | 80 (90.9%) | 8 (9.1%) |  |  |  |  |
| *few times/year* | 34 (100.0%) | 0 (0.0%) |  |  |  |  |
| **Decision to have children** |  |  |  |  |  |  |
| *Woman* | 31 (93.9%) | 2 (6.1%) | 0.618 |  |  |  |
| *Partner* | 15 (88.2%) | 2 (11.8%) |  |  |  |  |
| *both* | 282 (88.4%) | 37 (11.6%) |  |  |  |  |
| **Partner abuse** |  |  |  |  |  |  |
| *No* | 262 (86.5%) | 41 (13.5%) | **0.009** |  |  |  |
| *Yes* | 50 (100.0%) | 0 (0.0%) |  |  |  |  |
| *No answer* | 16 (100.0%) | 0 (0.0%) |  |  |  |  |
| **History of child abuse** |  |  |  |  |  |  |
| *No* | 273 (87.5%) | 39 (12.5%) | **0.047** |  |  |  |
| *Yes* | 55 (96.5%) | 2 (3.5%) |  |  |  |  |
| **Contraception use** |  |  |  |  |  |  |
| *No* | 82 (68.3%) | 38 (31.7%) | **0.01** |  |  |  |
| *Yes* | 30 (90.9%) | 3 (9.1%) |  |  |  |  |
| **Contraception method** |  |  |  |  |  |  |
| *Oral pills* | 13 (81.3%) | 3 (18.8%) | 0.217 |  |  |  |
| *IUD* | 5 (100.0%) | 0 (0.0%) |  |  |  |  |
| *Condoms* | 17 (100.0%) | 0 (0.0%) |  |  |  |  |
| *Other* | 3 (100.0%) | 0 (0.0%) |  |  |  |  |
| **Aware of emergency contraception (EC)** |  |  |  |  |  |  |
| *No* | 179 (88.2%) | 24 (11.8%) | 0.988 |  |  |  |
| *Yes* | 90 (88.2%) | 12 (11.8%) |  |  |  |  |
| **Reasons for not using contraception** |  |  |  |  |  |  |
| *Lack of knowledge* | 4 (100.0%) | 0 (0.0%) | **0.007** |  |  |  |
| *Fear of side effects* | 60 (88.2%) | 8 (11.8%) |  |  |  |  |
| *Religious concerns* | 1 (33.3%) | 2 (66.7%) |  |  |  |  |
| *Unavailable* | 1 (100.0%) | 0 (0.0%) |  |  |  |  |
| *Unaffordable* | 1 (100.0%) | 0 (0.0%) |  |  |  |  |
| *Desire to have more children* | 28 (60.9%) | 18 (39.1%) |  |  |  |  |
| *Other* | 11 (84.6%) | 2 (15.4%) |  |  |  |  |
