# Supplementary material for: Factors related to pregnancy status and unwanted pregnancy among lebanese women during the COVID-19 lockdown: a cross-sectional study
Source: Arch Public Health. 2022 Feb 25;80:68. doi: 10.1186/s13690-022-00833-2 (PMC8874299; doi:10.1186/s13690-022-00833-2)
Supplement: Supplementary file 2 — Additional file 2: Supplementary Table S2. Bivariate analysis taking the wanted/unwanted pregnancy as the dependent variable (N=41) [file 13690_2022_833_MOESM2_ESM.docx]

**Supplementary Tables:**

| **Supplementary Table 2: Bivariate analysis taking the wanted/unwanted pregnancy as the dependent variable (N=41)** | | | |
| --- | --- | --- | --- |
|  | **Wanted pregnancy**  **N=32 (78.0%)** | **Unwanted pregnancy**  **N=9 (22.0%)** | **p-value** |
| **Woman religion** |  |  |  |
| Christian | 13 (76.5%) | 4 (23.5%) | 1.000 |
| Muslim | 13 (76.5%) | 4 (23.5%) |  |
| Druze | 6 (85.7%) | 1 (14.3%) |  |
| **Woman working status** |  |  |  |
| Work from home | 5 (45.5%) | 6 (54.5%) | 0.032 |
| Going to work | 14 (93.3%) | 1 (6.7%) |  |
| Unemployed | 3 (100.0%) | 0 (0.0%) |  |
| Never work | 10 (83.3%) | 2 (16.7%) |  |
| **Woman income** |  |  |  |
| No income | 10 (76.9%) | 3 (23.1%) | 0.609 |
| Low | 6 (85.7%) | 1 (14.3%) |  |
| Intermediate | 14 (82.4%) | 3 (17.6%) |  |
| High | 2 (50.0%) | 2 (50.0%) |  |
| **Women smoking status** |  |  |  |
| Regular | 8 (72.7%) | 3 (27.3%) | 0.844 |
| Occasional | 9 (75.0%) | 3 (25.0%) |  |
| Previous | 1 (100.0%) | 0 (0.0%) |  |
| Non smoker | 14 (82.4%) | 3 (17.6%) |  |
| **History of unwanted pregnancy** |  |  |  |
| Yes | 1 (20.0%) | 4 (80.0%) | **0.006** |
| No | 31 (86.1%) | 5 (13.9%) |  |
| **Discussion with partner about family planning** | | | |
| Sometimes | 4 (50.0%) | 4 (50.0%) | **0.033** |
| Always | 28 (84.8%) | 5 (15.2%) |  |
| **Fertility preferences** |  |  |  |
| Desire to have children | 27 (87.1%) | 4 (12.9%) | **0.014** |
| Desire to stop childbearing | 5 (50.0%) | 5 (50.0%) |  |
| **Visiting regularly the physician** |  |  |  |
| Yes | 20 (87.0%) | 3 (13.0%) | **0.016** |
| No | 3 (42.9%) | 4 (57.1%) |  |
|  | **Mean (SD)** | **Mean (SD)** |  |
| **Women age in years** | 28.9 (5.3) | 29.9 (4.3) | 0.548 |
| **Age at marriage in years** | 25.6 (4.0) | 24.4 (4.6) | 0.477 |
| **Household crowding index** | 0.77 (0.32) | 1.2 (0.84) | 0.162 |
| **Fear of poverty** | 5.3 (3.4) | 5.1 (3.9) | 0.880 |
| **Number of children** | 1.3 (0.45) | 2.2 (0.83) | **0.002** |
| **CASR - total abuse scale** | 0.37 (1.3) | 0.44 (1.0) | 0.609 |
| CASR - Psychological abuse subscale | 0.25 (0.80) | 0.44 (1.0) | 0.588 |
| CASR - Physical abuse subscale | 0.12 (0.49) | 0.00 (0.00) | 0.793 |
| CASR - Sexual abuse subscale | 0.00 (0.00) | 0.00 (0.00) | 1.000 |
| *CASR: Composite Abuse Scale Revised; SD: standard deviation* | | | |
